# Supplementary material for: Impact of the Coronavirus Pandemic on High-Risk Infant Follow-Up (HRIF) Programs: A Survey of Academic Programs
Source: Children (Basel). 2021 Oct 6;8(10):889. doi: 10.3390/children8100889 (PMC8534718; doi:10.3390/children8100889)
Supplement: Supplementary file 1 [file children-08-00889-s001.zip › children-1386984-supplementary.pdf]

Current state of Neonatal High risk Follow clinic in era of coronavirus pandemic - A National Survey

You are being asked to take part in this research study because you are a practicing neonatologist and we are hoping to learn about how current coronavirus pandemic is affecting care in neonatal high risk follow up clinic (During the year 2020). There are minimal risks from this study, which include a possible breach of confidentiality. However, your responses are de-identified and cannot be linked back to you. Participation is voluntary and will take 10-15 minutes. Completion of this study implies consent. If you are a Texas Tech University Health Sciences El Paso's employee, your decision to participate will not be shared with your supervisor and will not have any effect on your performance evaluation or employment status. Please do not hesitate to contact us with questions by email (ajay.singh@ttuhsc.edu or sanjeet.panda@ttuhsc.edu) or phone (915-215-4987).

Does your program have A High-Risk Infant Follow-Up (HRIF) Clinic?

◯ Yes
◯ No

High Risk Neonatal Follow Up Clinic

Which of the following best describes Your current Role ?

◯ HRIF Clinic Director
◯ Neonatal-Perinatal Medicine Medical Director
◯ Division Chief (NICU)
◯ HRIF Clinic Coordinator
◯ Other

Please Specify

____

Please Specify Name of Hospital/University System With Which Your HRIF Clinic Is Affiliated With

____
(Please specify city and country of location)

Demographics of Your NICU

Number of Admissions in your NICU Per Year (averaged over last 3 years)

◯ < 250
◯ 251-500
◯ 501-750
◯ 751-1000
◯ >1000
(Per Year)

How Many Total Beds In NICU

____
(Total Beds)

High Risk Clinic

Current Criteria For Referral to HRIF clinic

☐ All NICU discharges
☐ All Less than 32 weeks Gestation or 1500 gram
☐ All Less than 36 weeks Gestation or 2500 gram
☐ Genetic anomalies
☐ HIE
☐ Seizures disorder
☐ Major Malformations(Medical or surgical)
☐ Others, please specify
(Check all that apply)

Other

____

What was the frequency of HRIF clinic at your program? (Pre-Pandemic)	◯ One half-day per week<br>◯ One full day per week<br>◯ Two full days per week<br>◯ More than two full days per week
Average Census Per HRIF Clinic Day (Pre-Pandemic)	◯ < 5<br>◯ 5-10<br>◯ 10-20<br>◯ >20
Did You Cancel High-Risk Clinic This year due to COVID Pandemic?	◯ No<br>◯ < 1 month<br>◯ 1-3 months<br>◯ 3-5 months<br>◯ >=6months
How Are You Currently Conducting Neonatal High Risk Follow Up Clinic Visits ?	◯ All In Person Visits<br>◯ All Telemedicine Visits<br>◯ Both In Person and Telemedicine Visit
Were your telemedicine visits Multidisciplinary (therapists, psychologist, dietitian) during pandemic?	◯ Yes<br>◯ no<br>◯ Other, Please comment
Other, Please Comment	__________________________________
Any Criteria That Would Mandate In Person Visit ?	☐ Developmental Testing<br>☐ Ventilator Dependency<br>☐ Oxygen Dependency<br>☐ TPN dependency ☐ Parental Requests<br>☐ New Complaints ☐ First OR Last HRIF Clinic Visit ☐ Other<br>(Choose All That Apply)
Please Specify Other	__________________________________
Do You Conduct Periodic Patient Satisfaction Survey Particular to High Risk Infant Follow Up Clinic	◯ Yes<br>◯ No
What Has The Reception Been To Changes Implemented To Neonatal High Risk Follow Up Clinic ?	Mostly Negative — Unchanged — Mostly Positive<br>*(Place a mark on the scale above)*
Any Changes To HRIF Clinic frequency Due To On Going Pandemic?	◯ Decreased Frequency of Clinic Visit Per Patient ◯ Increased Frequency Of Clinic Visit Per Patient ◯ No Change
What Changes Have You Noted In Clinic Show Rate During On-Going Pandemic	◯ Increased Show Rates<br>◯ Decreased Show Rates<br>◯ No Change In Show Rates

How Are You Approaching Measurement of Vitals For A Telemedicine Encounter ?

☐ Discharging Patients with Weighing Machine ☐ Discharging Patients with Measuring Tape ☐ Discharging Patients with Pulse Ox ☐ Asking Parents To Buy Their Own Equipment at Home ☐ Coordinating with Primary Care Provider to Obtain Vital from those visits ☐ No Vitals Measured or Asked For ☐ Other Method
(Choose All That Apply)

Please Specify, Other Method

____

Personnel Available in Clinic (Choose all that apply, even if not on all clinic days)

	Were Never Part Of Clinic	Available Pre Pandemic	Available During Pandemic
Neonatologist	☐	☐	☐
Advance Nurse Practitioner	☐	☐	☐
Registered Nurse	☐	☐	☐
Physical Therapist	☐	☐	☐
Occupational Therapist	☐	☐	☐
Speech Therapist	☐	☐	☐
Nutritionist	☐	☐	☐
Developmental Specialist	☐	☐	☐
General Pediatrician	☐	☐	☐

How Are State Funded Therapy Programs Currently Being Administered in Your Area for high risk infants ? (Select Both If Applicable)

	Virtually	In Person
Physical Therapy	☐	☐
Occupational Therapy	☐	☐
Speech Therapy	☐	☐
Other Therapies	☐	☐

Perception of Clinic

Did Reimbursement/payor policies affect your decision to conduct HRIF clinics in person vs telemedicine

◯ Yes, led to do more telemedicine visits
◯ Yes, led to do more in person visits
◯ No, did not affect my decision

Did you develop a method to standardize your telemedicine visits?

◯ Yes, please comment
◯ No

Please choose all, that was standardized

☐ Intake, and history taking.
☐ Physical Exam
☐ Developmental screening/testing, please specify
(multiple choice)

Please enumerate all the Developmental screening or Testing (eg. ASQs, HINES) that was standardized for telemedicine visits.

____

What was level of reimbursement for Telemedicine visits, compared to in-person visits.

(Place a mark on the scale above)

What Is Your Perception Of Changes in High Risk Infant Clinic Format ?

Mostly Negative | Neutral | Mostly Positive

(Place a mark on the scale above)

Was your HRIF clinic able to conduct home visits during pandemic ?

☐ Yes ☐ No ☐ Home visits were never part of HRIF Clinic

Any comments or feedback you would like to share.

____

Short Follow-up Survey

Thanks for responding to the initial survey, as per feedback from our initial survey responders, we have added 6 more questions to make the survey more useful. You are being asked to take part in this research study because you are a practicing neonatologist and we are hoping to learn about how the current coronavirus pandemic is affecting care in neonatal high risk follow up clinic (During the year 2020).

Please do not hesitate to contact us with questions by email (ajay.singh@ttuhsc.edu or sanjeet.panda@ttuhsc.edu) or phone (915-215-4987).

Were your telemedicine visits Multidisciplinary (therapists, psychologist, dietitian) during pandemic?	◯ Yes<br>◯ no<br>◯ Other, Please comment
Other, Please Comment	__________________________________
Did Reimbursement/payor policies affect your decision to conduct HRIF clinics in person vs telemedicine	◯ Yes, led to do more telemedicine visits<br>◯ Yes, led to do more in person visits<br>◯ No, did not affect my decision
Did you develop a method to standardize your telemedicine visits?	◯ Yes, please comment<br>◯ No
Please choose all, that was standardized	☐ Intake, and history taking.<br>☐ Physical Exam<br>☐ Developmental screening/testing, please specify<br>(multiple choice)
Please enumerate all the Developmental screening or Testing (eg. ASQs, HINES) that was standardized for telemedicine visits.	__________________________________
What was level of reimbursement for Telemedicine visits, compared to in-person visits.	*(Place a mark on the scale above)*
Was your HRIF clinic able to conduct home visits during pandemic ?	☐ Yes ☐ No ☐ Home visits were never part of HRIF Clinic
Any comments or feedback you would like to share.	__________________________________
